# Prevalence of violent advertisements in New York City subways

**DOI:** 10.34172/hpp.2021.27

**Published:** 2021-05-19

**Authors:** Dottington Fullwood, Carrie Cameron, Sydney Means, Stephen Anton, Zachary L. Stickley, Randal Hale, Diana J. Wilkie

**Affiliations:** ^1^Department of Aging and Geriatric Research, College of Medicine, The University of Florida, Gainesville, Florida, 32611, USA; ^2^Division of Cancer Prevention and Population Sciences, Cancer Prevention Research Training Program, The University of Texas MD Anderson Cancer Center, Houston, Texas, 77230, USA; ^3^Department of Educational Psychology and Leadership, Texas Tech University, Lubbock, Texas, 77030, USA; ^4^North River Geographic Systems, Inc., Chattanooga, TN, 37411, USA; ^5^Department of Biobehavioral Nursing Science, College of Nursing, University of Florida, Gainesville, Florida, 32610, USA

**Keywords:** Advertising, Aggression, New York City, Subways, Violence

## Abstract

**Background:** Media advertisements displaying aggression and violence in public transit spaces represent a public health concern. The high visibility of ads likely contributes to increased levels of aggression among New York City (NYC) youths traveling across boroughs. Given the importance of the physical, psychological and social environment in shaping the lives of youth, additional attention is warranted regarding how media advertisements are promoted within public transit spaces across America. The aim of this study was to document quantity and placement of advertisements illustrating aggressive and violent content throughout the NYC public transit subway system.

**Methods:** This cross-sectional study was conducted over a five-day period in June 2017. Direct observation was used to document all advertisements within every NYC Metropolitan Transit Authority (MTA) subway station (N = 472) in four NYC boroughs: Bronx, Brooklyn, Manhattan and Queens. Static media advertisements with/without aggressive and violent content displayed on subway platform wall panels above and underground were counted, photographed and described with a mobile app.

**Results:** Aggressive and violent ads in the MTA were pervasive. Subway platforms displayed advertising consisting of guns, individuals fighting and attacking, and words with aggressive language.

**Conclusion:** Public transit spaces provide unregulated visual and verbal messages without citizen participation. Subway stations in NYC and across the country prohibition stance could be a model for violent content reduction. Given the pervasive and tragic effects of aggression and violence on youth and adults, transit agencies could inundate passengers with positive advertising content. Dialogue between citizens and transit agencies to remove noxious messages from public transit spaces warrants the same discussion given to banning alcohol advertisements.

## Introduction

Media is a pervasive and important influence on the environment because of its reach and potential harmful effects. Perhaps one of its most harmful effects is the creation of high levels of acceptability of aggression among youth, which may be generated by the high concentration of advertisements that promote aggressive portrayals and depict forms of violent behavior as part of their advertising message. Advertisers effectively hold consumer attention by using message cues with aggressive images and words targeted at vulnerable audiences.^1^ Message cues can be thought of as cognitive scripts that may develop into steadfast memorable moments that result from constant exposure to violent content that could eventually translate into aggression expressed in social situations.^2^

Public transportation for many children and adults is a resource utilized to arrive at school and work. Individuals younger than age 25 account for more than 20% of all public transit riders in the United States.^3^ New York City (NYC) operates the largest public Metropolitan Transportation Authority (MTA) subway system in the U.S. and serves the five boroughs of NYC. Approximately 600,000 Metrocards (e.g., subway tokens) are distributed by the NYC MTA each year to students living in NYC. These stations are also covered in advertising, with many companies vying for the business of these metropolitan consumers. However, the amount of exposure to advertisements urban subway riders encounter and how these messages affect public health has largely gone unexplored.

While using this form of public transportation, the potential corporate advertising exposure for students may increase the immediate and distal effects of aggressive and violent ads that extend beyond brand identification to real life trauma as a result of aggressive and violent actions. For example, according to the Information Processing Model, a movie advertisement depicting a gun is not likely to be purposefully evaluated but is rather processed implicitly and acts as an associative learning stimulus, which activates positive attitudes toward the source.^4-7^ Research suggests that favorable attitude formation toward a brand can form from mere exposure coupled with associative learning.^6^

The social and cultural contexts of acts of aggression and violence in places where people live and interact represent the social environment that may shape these behaviors. For this reason, there has been growing effort of advocacy across the U.S. for removal of violent media advertisements from public spaces. Such calls to action are in line with the Information Processing Model, which asserts formation of memorable “cognitive scripts” resulting from constant exposure to violent content provoke aggressive behavior instead of inhibiting aggression in social situations.^2^ The impact and magnitude of aggressive and violent media content on the actions of both youths and adults remain unknown, but it is likely to be destructive.

The present study sought to document the nature and scope of advertising throughout the NYC subway system, which is used by nearly two million New Yorkers,^8^ with specific emphasis on the portrayals of ads/advertisements displaying aggressive and violent content.

## Materials and Methods

This cross sectional study was built on findings of a small pilot study that demonstrated proof of need for a larger study with systematic methods.^9^ The study was limited in scope to single stations and station complexes within four NYC boroughs, including Bronx, Brooklyn, Manhattan and Queens. Single stations were defined as those with only one train line, (i.e., local trains) and with no passageways to other train lines. Station complexes were defined as stations serving multiple train lines, which may or may not connect to different train lines. To identify the type and percentage of aggressive and violent advertisements within NYC, a research team member visited both single stations and station complexes operated in the NYC MTA over a five-day period in June 2017.

The multiple points of subway entrances required a systematic approach of surveillance to detail the presence of a particular appeal. This study focused on static advertisements on platform wall panels distributed throughout the MTA single stations and station complexes. These static two-sheet large platform billboards are 60 inches wide by 46 high and attract an estimated 20,000 impressions (i.e., number of eyeballs the ad attracts) per advertisement per day with a 25% recommended reach of the daily population.^10^ We documented 143 stations above and underground that contained one or more subway platform advertisements on the walls. We surveyed the wall space and passageways toward other subway platforms that potentially offered continuous media advertisements for the length of the passageway.

### Inclusion and exclusion criteria

All advertisements located on the subway platform walls of every subway station line were included. Stations that had advertisements on the walls outside of the turnstile heading toward the enter/exit stairwell were assessed as well. All digital kiosks that displayed brief rotating ads and MTA service announcements were excluded for minimal display time. As mentioned above, stations identified as station complexes included subway platforms as well as every passageway that may have potentially displayed advertisements. Subway stations that displayed no advertisements on the subway platform were documented so that the number and location of these stations could be incorporated into the analysis. MTA advertisements located within the entryway to subway stations were excluded because of multiple points of station entry. The distribution of turnstile advertisements was low throughout the four boroughs and therefore excluded from the data collection. All advertisements that were partially removed in such a way that coding could not be completed were excluded. The number of these defaced advertisements was documented.

### Data collection

**Figure 1:**
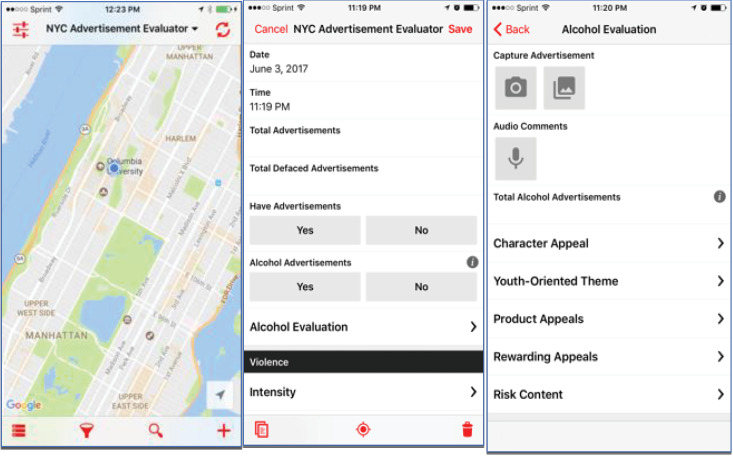
NYC Advertisement Evaluator-Fulcrum Mobile App.

Using a mobile data collection app (Fulcrum mobile location app; Figure 1) allowed for collection of subway stop details and captured a photograph of the advertisement on the platform. The Fulcrum mobile location app has cloud-based functionality with unlimited storage capacity that provides real-time iOS and Android mobile data collection with geographic information system (GIS) anywhere and anytime. We customized the app (termed *NYC Subway Inspection App*) to collect predefined choice lists of attributes about the subway stop and information about the characteristics of the advertised media content as well as date, latitude, longitude, time, whether the platform had an advertisement, was a single or complex station, had defaced advertisement, photo of advertisement, subway line, and station location stored for later data retrieval from a web-based cloud server. These customized lists allowed for input of descriptive content about the advertisement based on the photographs. The information collected was stored on the mobile device and synced data to the Fulcrum application programming interface (i.e., internet cloud).

Data collection using Fulcrum to record every advertisement at single stations and station complexes occurred over a five-day period in June 2017 for every train line operating in the Bronx, Brooklyn, Manhattan, and Queens. The lead team member rode the MTA Blue train line (A train) toward Far-Rockaway-Mott station (end of the line) exiting at each subway stop to count and photograph all advertisements on the platform for that particular subway stop and board the following train to the next stop and apply the same procedure until the Far-Rockaway destination was reached. At the end of the line, the investigator either crossed the platform or exited the station in order to ride the train line in the other direction toward 207^th^ Street subway stop. The same method of advertisement documentation occurred at every subway stop until the investigator arrived at the last stop for that train line. Upon completion of a particular downtown and uptown train line, the investigator navigated to the next train line and applied the same method of data collection. Every station complex was inspected for advertisements located along the wall space of every passageway that led toward another transfer platform. The surveillance of these advertisements within these complexes were counted as one station.

### Coding of advertisements 

Advertisements located on the downtown or uptown side of the platform for each station were documented. Aggressive or violent content advertisements after data collection were reviewed independently by three team members. Each team member categorized advertisements as present or absent of the following themes: animated characters, guns, weapons other than guns, images of fighting/attacking, fear, anger/aggression, destruction, injured people, and words associated with aggression and violence. For any items in which there was disagreement among the raters, these themes were discussed among the three team members until consensus was developed about the correct categorization of each identified aggressive or violent advertisement.

### Data analyses

The primary aim of this census study involved calculating descriptive statistics including frequencies and percentages of the distributions by borough and for all four boroughs combined regarding the total number of stations, with and without any advertisements, violent advertisements, as well as for the absolute number of advertisements with violent content. A variety of methods were used to determine the proportion of total advertisements with violent content and the proportion of total stations with and without advertisements with violent content. For advertisements with violent content, the frequencies and percentages were calculated regarding the object (i.e., movie, television show, play, or public service), and then each advertisement was categorized as violent if any of the following images were present: animation, guns, weapons other than guns, images of fighting/attacking, fear, anger/aggression, destruction, injured people and words associated with aggression and violence. All analyses were conducted in R version 3.3.1.^11^

## Results

**Figure 2:**
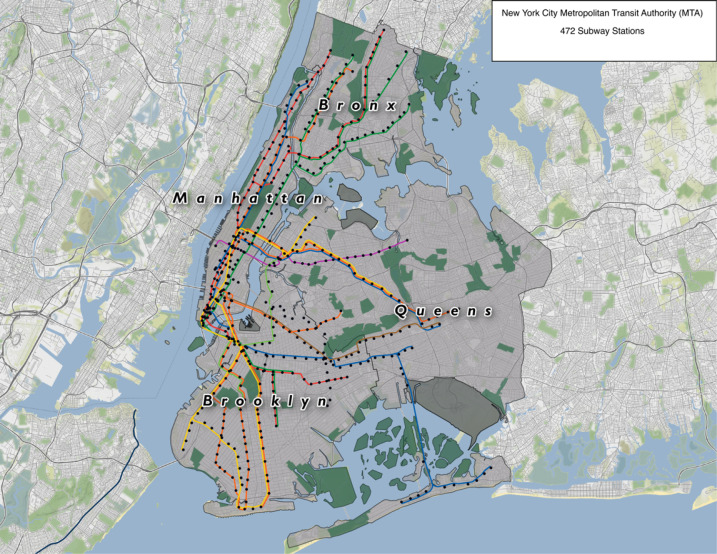
NYC Metropolitan Transit Authority subway stations within the four boroughs.

There was a total of 472 subway stations (Figure 2) with and without advertisements, summarized in Table 1 across four observed boroughs. Of these, 143 (30.3%) contained advertisements. We documented 8737 advertisements, including duplicates. Brooklyn had the greatest number of both stations (169) and advertisements (3100) followed by Manhattan (151 stations; 2751 advertisements), Queens (82 stations; 1834 advertisements) and Bronx (70 stations; 1052 advertisements).

The percentage of stations with advertisements ranged from a low of 25.4% in the Brooklyn (43/169) to a high of 35.1% in Manhattan (53/151). Of the stations with advertisements, over 95% (136/143) had one or more advertisements with violent content. There was minimal variation in the rate of stations with violent advertisements (stations with violent advertisements/stations with any advertisements), with over 90% of the stations in every borough that had advertisements displaying one or more with violent content.

**Figure 3:**
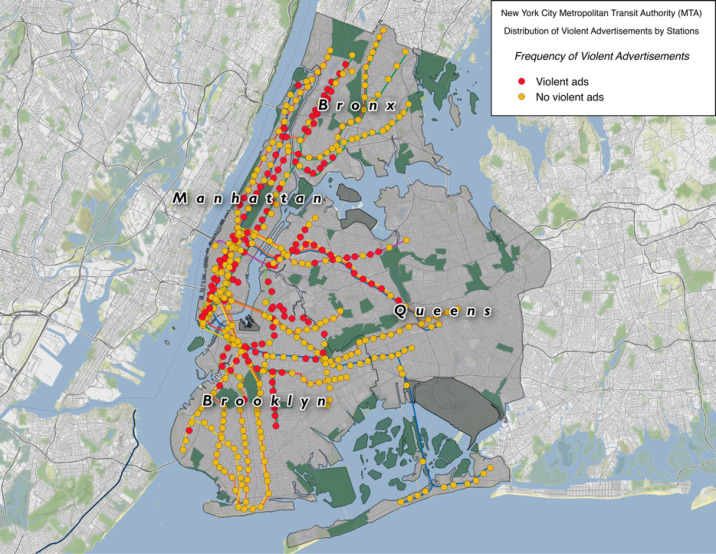
NYC Metropolitan Transit Authority frequency of violent ads per station.

Of the 8737 advertisements observed, 1154 (13.2%) were for violent content. The number of advertisements with violent content in the boroughs ranged from 115 in the Bronx to 425 in Manhattan (Figure 3). The percentage of all advertisements within the boroughs that displayed violent content ranged from 11% in the Bronx (115/1,052) to 15.4% in Manhattan (425/2,751).

There was considerable variability between the boroughs. Even though Brooklyn had the lowest rate of stations with advertisements (i.e., number of stations with advertisements/total number of stations), Brooklyn had the second highest absolute number of stations with advertisements (n = 43). Manhattan and Brooklyn had the highest absolute number of stations with violent advertisements, which mirrored the larger number of stations both with and without advertising in these boroughs.

### Bronx

The Bronx is serviced by 70 NYC subway stations in a total of 28 neighborhoods, with the number of stations per neighborhoods varying from one (n = 6) to seven (n = 1), and a majority of neighborhoods having two (n = 8) or three (n = 9) (Table 2) stations. Eleven percent of the 1052 total advertisements documented in the Bronx (n = 116) included violent content and were observed in 10 of the 11 neighborhoods with advertisements, which included 20 stations. Eighty-one percent of all the advertisements in the Bronx were concentrated within 5 of the 28 neighborhoods (Table 2; n = 851/1052), accounting for 75% of the violent advertisements (n = 86/115). Excluding the neighborhoods with no advertisements, the prevalence rate of violent advertisements across the neighborhoods (i.e., number of violent advertisements/total advertisements) varied from a low of 5% (1/20) to a high of 23.3% (14/60).

### Brooklyn

Brooklyn comprises 44 neighborhoods serviced by 169 subway stations, with the largest number of neighborhoods and stations of any borough in NYC, the largest number of total advertisements (n = 3100), and a correspondingly large number of advertisements with violent content (n = 404; Table 3). These advertisements were present in all 23 neighborhoods serviced by 43 stations, 25.4% of the total number of stations across Brooklyn. Approximately one-half of the total number of advertisements (1537/3,100) were located in six neighborhoods (Table 3). While these neighborhoods comprised only 18.3% of the total number of stations in Brooklyn (31/169), they included almost one-half of the violent advertisements (184/404, 45.5%).

### Manhattan

A total of 151 subway stations were observed in 28 neighborhoods located in the borough of Manhattan (Table 4) ranging from 1 to 22 per neighborhood. Four of the 28 neighborhoods accounted for 38.4% (1057/2,751) of all the advertisements displayed in Manhattan. Among 21 of the 22 neighborhoods containing 48 of 151 stations (32%) with advertisements, >95% displayed advertisements with violent content. More than two-thirds of 425, violent ads (209; 68.2%) were displayed in 8 of the 28 neighborhoods, 3 of which accounted for over 30% of the total number of advertisements with violent content (n = 137/425; 32.2%; Table 4).

### Queens

There are 82 subway stations dispersed throughout the 24 neighborhoods in Queens (Table 5). Twenty three of the 24 neighborhoods (95.8%), and 81 of the 82 stations (98.8%), displayed advertising with violent content. Of the 1834 advertisements observed in Queens, 210 included violent content (11.5%).

### Identifying violent content

Each of the 45 unique advertisements with violent content was coded with respect to the presence of particular attributes defined by the research team. Images of anger and aggression were the most commonly observed category, present in over 70% of the advertisements (n = 813) (Table 6). The second most frequently observed category was words associated with aggression or violence (e.g., ‘fear’, ‘pain’, ‘blood sucker’, ‘ass kicker’). Images of fear such as loaded gun pointed directly at the viewer were present in almost one-half of the advertisements (n = 540). Of the 1154 advertisements with violent content, more than 1 in 4 depicted animation characters shipwrecked with sharp spears attacking one another for survival (304, 26.3%). Another 144 advertisements pictured guns (12.5%) and 437 (37.9%) pictured weapons other than guns. None of these advertisements depicted prevention of aggressive behavior or violent acts.

## Discussion

To our knowledge, this was the first study to document the pervasiveness of advertisements using aggressive and violent content throughout over 450 stations of New York MTA subway system. This study also shows that this content is not equally applied in advertising throughout the different stations, but rather different boroughs appear to be subjected to more advertisements with aggressive and violent themes than others, and even within each borough the variance in amount of aggressive or violent themed advertisements seems to be high. While the impact of these advertisements is ideally for the casual observer to gain an interest in what is being advertised, this study draws attention to the need to study the impact of the pervasive presence of aggressive and violent imagery in our society.

Aggression and violence in both media and real life is pervasive in America in general and among youth in particular. For example, the Youth Risk Behavior Surveillance System,^12^ an ongoing biennial survey of American high school students, showed that, during a 30-day period preceding the survey, over 16% carried a gun, knife, or club on at least one day, 6% were threatened or injured with a weapon on school property, and more than 5% did not go to school because of safety concerns. Also during a 12-month period preceding the survey, more than 20% were in a physical fight and more than 20% were bullied on school property.^12^ Homicide is the third leading cause of death for youth aged 1-4 and 15-24 years and the fourth leading cause of death for youth between the ages of 5 and 14 years.^13^ While the association between portrayals of media violence and lived experience of youth will continue to be debated, it seems clear that exposing youth to images and words depicting fear, anger, guns and other weapons does not help cultivate a social and psychological environment epitomizing a healthy community.^14-16^

Of note, there was not a single advertisement observed on the platforms that was related to violence prevention, conflict resolution, or community. This finding is unfortunate since communications on subway platforms provide an opportunity to reach many people with positive messages on a daily basis. Although such communications may not generate advertising revenue, they can help cultivate a positive social and psychological climate.

This study demonstrated that advertising content, in particular violent content, is far from evenly distributed throughout the NYC subway system. Citizens that live and grow in these unequal environments are likely to be affected in ways that influence their thoughts and feelings.^17-19^ The emotional toll of these lived experiences may affect each person in ways that lead to desensitization and thinking about this form of advertised communication as normal. These identified advertisements may have promoted anger and/or aggressive violent content. Thus, neighborhoods with high concentration of people of color and poverty and are more likely to be exposed to advertising in general and to advertising with aggressive and/or violent content. Taken together, exposures such as these impact neurologic synaptic pruning processes that are highly influential across the life span.^17,20^ Therefore, it is important that future research considers the powerful effects of the social environment, including how media advertisements may influence adolescent development.^21,22^

Of the 1154 advertisements with violent content, more than one in four (n = 304) contained animated caricatures. This is troubling since these kinds of images are likely to attract attention from children, a tactic adopted by the tobacco industry with their use of “Joe Camel.” The repeated exposures may prove vital for a growing child that may utilize this information to guide behavior during problem-solving when resolving conflict.^2,23^

The number of images with guns promoting violence, especially those including guns, is troubling. A majority of the violent products reviewed displayed images of anger and or aggression with most highlighting weapons portraying violent intent to harm. NYC Health Department created an initiative with a focus to stop the spread of violence.^24^ Given the magnitude and severity of gun violence and systemic racism in America, exposing youth and adults to such images is destructive.^25-31^ The findings of this research indicate a misalignment between that goal and the hundreds of noxious images that promote aggression and violence on subway platforms traveled by thousands of New Yorkers, including children and adolescents, on a regular basis. Government advocacy efforts outlined to interrupt conflicts and change social norms to improve community^24^ may benefit from surveying citizens about the high risk associated with aggressive words or acts of violence displayed on advertisements in public spaces travelled by many citizens on a daily basis.

### Strengths and Limitations

The Fulcrum mobile app provide a novel electronic method compared to paper and pencil. Data collection in real-time led to effective and efficient documenting of thousands of images in a reliable manner.

Our study provides a baseline reference snap-shot that reveals a seasonal view of advertisement placement across subway stations. Longitudinal work could augment the findings with an emphasis focused on neighborhood characteristics including crime statistics and racial compositions. Additionally, the use of more than one coder to determine what constitutes an advertisement as having violent content during initial coding may have possibly included or excluded advertisement deemed acceptable by another coder. However, two other investigators reviewed all of the advertisements with violent content and agreed that they warranted inclusion in the sample; thus, we believe the sample portrays an accurate representation of aggressive and violent advertisements exhibited on the subway platforms of NYC. Further collection of advertisements displayed in other subway areas such as kiosks, turnstiles, and entry and exit points in the subway would add to the broader picture of prevalence of violent content.

### Implications for policy and citizen engagement

The City of New York Mayor’s office crafted a blueprint, Take Care New York 2020, designed to improve community health for everyone by tackling the top health priorities and making NYC more equitable. Feedback from over 1,000 community consultants from every borough except Manhattan listed violence as a top priority for change.^24^ Given that so many of the subway stations observed in our study displayed images of guns, individuals fighting and attacking, and language associated with violence, subway advertising would be a good place to start changing NYC’s environment. In October 2017, the board of the MTA banned advertisements for alcohol on buses, trains and stations based on the idea that the advantages of discouraging underage drinking outweighed the loss of revenue.^32^ Given the pervasive and tragic effects of aggression and violence on youth (and adults), the MTS board should consider extending their logic to other forms of advertising. There is a need for New Yorkers to become engaged in this issue and take a stand about the kinds of images and communications that are exhibited in ‘their’ public spaces.

Artists and producers have the right to depict aggression and violence in privately consumed television, movies and other forms. Individuals choosing to watch a particular movie or television show are making a choice. Some efforts have been made to restrict exposure of youth to such media, for example through ratings assigned to movies. With respect to transit advertising, commuters trying to get to and from their workplace or students traveling to and from school have no choice. Citizens are inundated with a plethora of images and words associated with entertainment prescribed as action, adventure, crime, drama, fantasy, horror, thriller, and war, over which they have no control. Is this how we, as citizens, want to use our public spaces?

Citizens who engage with these screen time novelties may view the destructive, harmful and weapon-loaded imagery as a form of unrealistic escapism or a form of entertainment. The relief of escapism fails to diminish the harsh realities experienced by children, adolescents and adults of color living in environments with constant and random acts of aggression and violence, and various studies suggest that exposure to aggression or violence often translates into unfavorable outcomes such as unintentional injury, interpersonal murder or self-harm.^2,18,19,33^

### Recommendations for future research

Public transportation (i.e., subway) is an important life path point through which so many people travel on a daily basis. Research investigating subway platform wall advertising remains sparse across America’s subway systems. Media advertisements displayed in public spaces warrant ongoing surveillance. Surveillance audits at multiple time points will help document the nature and extent of advertisements in corridors and platforms that reach tens of thousands of people in NYC alone each day. The social environment plays an influential role in shaping health and human behavior. Research and advocacy to address the issue of aggressive and violent public advertising can help to promote positive behaviors.

## Acknowledgments

The authors would like to thank Drs. Wallace, Carrera, Basch and Basch, Valerie Marchenko and Pam Howard. for their assistance with data analysis and technical editing of this manuscript. Dr. Fullwood was supported by the Jacksonville Aging Studies Center (NIA 3R33AG056540-04S2) and the University of Florida’s Claude D. Pepper Older Americans Center (NIH/NIA P30AG028740).

## Funding

This project has no funding to declare.

## Competing interests

The authors declare that they have no known financial interests or personal relationships that could have appeared to influence the work reported in this manuscript. The content is solely the responsibility of the authors and does not necessarily represent the official views of the National Institutes of Health or other funding agencies.

## Ethical approval

The study protocol (Protocol # 17-335) submitted to the Institutional Review Board (IRB), at Teachers College, Columbia University determined this study exempt because the project does not involve human subjects.

## Authors’ contributions

DF: Conceptualization, data curation, formal analysis, writing-original draft preparation, Writing-reviewing and editing. CC, SM, SA and DJW: Writing-reviewing and editing. ZLS: Formal analysis, writing-reviewing and editing. RH: Data curation, formal Analysis.

**Table 1:** Frequencies and rates of advertisements, subway stations with and without advertisements, and type of advertisements by Borough, New York City

	**Bronx**	**Brooklyn**	**Manhattan**	**Queens**	**Total**
Total Ads	1052	3100	2751	1834	8737
Total stations	70	169	151	82	472
Stations with Ads	21	43	53	26	143
Stations without Ads	49	126	98	56	329
Stations with violence Ads	20	43	48	25	136
Number of violence Ads	115	404	425	210	1154
Rate of violent Ads/total Ads	11.0	13.0	15.4	11.5	13.2
Rate of Stations with violence Ads when any ads are present	95.2	100	90.6	96.2	95.1

The rate and total number of advertisements were based on stations located in four NYC boroughs. These remaining values were primarily based on field collection data regarding violent and other advertisements.

**Table 2:** Frequencies of subway stations with and without any advertisements, and advertisements about alcohol and violent content, and frequencies and rates of advertisements about alcohol and violence, Bronx, New York City

**Neighborhood**	**Stations**					**Advertisements**			**Rates**	
	**Total**	**Ads**	**No Ad**	**Alc Ad**	**Vio Ad**	**Alc**	**Vio**	**Total**	**Alc**	**Vio**
Allerton-Pelham Gardens	2	0	2	0	0	0	0	0	0	0
Bedford Park	4	2	2	2	2	3	6	76	3.9	7.9
Bronxdale	2	0	2	0	0	0	0	0	0	0
Co-op City	1	0	1	0	0	0	0	0	0	0
Crotona Park East	1	0	1	0	0	0	0	0	0	0
East Concourse	1	1	0	1	1	2	16	128	1.6	13.3
East Tremont	3	0	3	0	0	0	0	0	0	0
Eastchester-Edenwald	0	1	0	0	0	0	0	0	0	0
Fordham South	2	2	0	1	2	3	20	190	1.6	10.5
Hunts Point	3	0	3	0	0	0	0	0	0	0
Longwood	3	2	1	1	2	1	14	60	1.7	23.3
Melrose South	3	0	3	0	0	0	0	0	0	0
Morrisania-Melrose	1	0	1	0	0	0	0	0	0	0
Mott Haven-Port Morris	7	5	2	2	5	3	22	148	1.4	14.9
Mount Hope	3	2	1	1	1	5	12	165	2.4	7.3
Norwood	3	1	2	1	1	1	3	15	6.7	20.0
Pelham Bay	3	0	3	0	0	0	0	0	0	0
Pelham Parkway	2	0	2	0	0	0	0	0	0	0
Soundview-Bruckner	3	0	3	0	0	0	0	0	0	0
Spuyten Duyvil	3	0	3	0	0	0	0	0	0	0
University Heights	3	1	1	0	1	0	1	20	0	5.0
Van Cortlandt Village	2	2	0	1	2	2	5	30	6.7	16.7
Van Nest-Morris Park	1	0	1	0	0	0	0	0	0	0
West Concourse	5	2	3	2	3	3	16	220	1.4	7.3
West Farms-Bronx River	1	0	1	0	0	0	0	0	0	0
Westchester-Unionport	2	0	2	0	0	0	0	0	0	0
Williamsbridg-Olinville	4	0	4	0	0	0	0	0	0	0
Woodlawn-Wakefield	2	0	2	0	0	0	0	0	0	0
**Totals**	**70**	**21**	**49**	**12**	**20**	**23**	**115**	**1052**		

Alc Ad, Alcohol Advertisement; Vio Ad, Violent Advertisement.

**Table 3:** Frequencies of Subway Stations with and Without Any Advertisements, and Advertisements About Aggressive and Violent Content, and Frequencies and Rates of Advertisements about Violence, Brooklyn, New York City.^1^

**Neighborhood**	**Stations**					**Advertisements**			**Rate**	
	**Total**	**Ads**	**No Ad**	**Alc Ad**	**Vio Ad**	**Alc**	**Vio**	**Total**	**Alc**	**Vio**
Battery Park City Lower	14	4	10	0	3	0	33	165	0	20.0
Central Harlem North	4	2	2	2	2	5	18	146	3.4	12.3
Central Harlem South	5	4	1	3	3	4	27	162	2.5	16.7
Chinatown	5	1	4	1	1	1	12	72	1.4	16.7
Clinton	1	1	0	0	1	0	10	35	0	28.6
East Harlem North	2	0	2	0	0	0	0	0	0	0
East Harlem South	3	1	2	0	1	0	5	19	0	26.3
East Village	2	1	1	0	1	0	9	57	0	15.8
Gramercy	4	3	1	1	3	1	19	170	0.6	11.2
Hamilton Heights	3	1	2	1	1	4	12	119	3.4	10.1
Hudson Yards-Chelsea	10	3	7	1	3	1	32	168	0.6	19.0
Lenox Hill	2	0	2	0	0	0	0	0	0	0
Lincoln Square	4	2	2	1	0	1	0	33	3.6	0
Lower East Side	1	0	1	0	0	0	0	0	0	0
Manhattanville	2	1	1	1	1	2	8	56	1.1	15.4
Marble Hill-Inwood	4	0	4	0	0	0	0	0	0	0
Midtown-Midtown South	22	6	16	2	6	4	57	369	0	6.9
Morningside Heights	4	0	4	0	0	0	0	0	0	0
Murray Hill-Kips Bay	1	1	0	0	1	0	2	29	0	6.9
SoHo TriBeCa	17	6	11	1	5	1	39	251	0.4	15.5
Turtle Bay-East Midtown	3	1	2	0	1	0	3	20	0	15.0
Upper East Side	7	4	3	0	4	0	41	194	0	21.1
Upper West Side	5	3	2	0	3	0	27	141	0	19.1
Washington Heights North	5	1	4	1	1	2	13	116	1.7	11.2
Washington Heights South	5	1	4	1	1	2	10	108	1.9	9.3
West Village	9	2	7	1	2	2	14	78	2.6	17.9
Yorkville	1	0	1	0	0	0	0	0	0	0
Park-Cemetery-etc.	6	4	2	1	4	2	34	243	0.8	14.0
**Totals**	**151**	**53**	**98**	**18**	**48**	**32**	**425**	**2751**		

Alc Ad, Alcohol Advertisement; Vio Ad, Violent Advertisement.

**Table 4:** Frequencies of subway stations with and without any advertisements, and advertisements about alcohol and violent content, and frequencies and rates of advertisements about alcohol and violence, Manhattan, New York City.

**Neighborhood**	**Stations**					**Advertisements**			**Rate**	
	**Total**	**Ads**	**No Ad**	**Alc Ad**	**Vio Ad**	**Alc**	**Vio**	**Total**	**Alc**	**Vio**
Battery Park City Lower	14	4	10	0	3	0	33	165	0	20.0
Central Harlem North	4	2	2	2	2	5	18	146	3.4	12.3
Central Harlem South	5	4	1	3	3	4	27	162	2.5	16.7
Chinatown	5	1	4	1	1	1	12	72	1.4	16.7
Clinton	1	1	0	0	1	0	10	35	0	28.6
East Harlem North	2	0	2	0	0	0	0	0	0	0
East Harlem South	3	1	2	0	1	0	5	19	0	26.3
East Village	2	1	1	0	1	0	9	57	0	15.8
Gramercy	4	3	1	1	3	1	19	170	0.6	11.2
Hamilton Heights	3	1	2	1	1	4	12	119	3.4	10.1
Hudson Yards-Chelsea	10	3	7	1	3	1	32	168	0.6	19.0
Lenox Hill	2	0	2	0	0	0	0	0	0	0
Lincoln Square	4	2	2	1	0	1	0	33	3.6	0
Lower East Side	1	0	1	0	0	0	0	0	0	0
Manhattanville	2	1	1	1	1	2	8	56	1.1	15.4
Marble Hill-Inwood	4	0	4	0	0	0	0	0	0	0
Midtown-Midtown South	22	6	16	2	6	4	57	369	0	6.9
Morningside Heights	4	0	4	0	0	0	0	0	0	0
Murray Hill-Kips Bay	1	1	0	0	1	0	2	29	0	6.9
SoHo TriBeCa	17	6	11	1	5	1	39	251	0.4	15.5
Turtle Bay-East Midtown	3	1	2	0	1	0	3	20	0	15.0
Upper East Side	7	4	3	0	4	0	41	194	0	21.1
Upper West Side	5	3	2	0	3	0	27	141	0	19.1
Washington Heights North	5	1	4	1	1	2	13	116	1.7	11.2
Washington Heights South	5	1	4	1	1	2	10	108	1.9	9.3
West Village	9	2	7	1	2	2	14	78	2.6	17.9
Yorkville	1	0	1	0	0	0	0	0	0	0
Park-Cemetery-etc.	6	4	2	1	4	2	34	243	0.8	14.0
**Totals**	**151**	**53**	**98**	**18**	**48**	**32**	**425**	**2751**		

Alc Ad, Alcohol Advertisement; Vio Ad, Violent Advertisement.

**Table 5:** Frequencies of subway stations with and without any advertisements, and advertisements about alcohol and violent content, and frequencies and rates of advertisements about alcohol and violence, Queens, New York City.

**Neighborhood**	**Stations**					**Advertisements**			**Rate**	
	**Total**	**Ads**	**No Ad**	**Alc Ad**	**Vio Ad**	**Alc**	**Vio**	**Total**	**Alc**	**Vio**
Astoria	5	5	0	4	5	8	37	267	3.0	13.9
Breezy Point-Belle Harbor	4	0	4	0	0	0	0	0	0	0
Briarwood-Jamaica Hills	1	0	1	0	0	0	0	0	0	0
Elmhurst	6	3	3	3	3	6	34	377	1.6	9.0
Far Rockaway-Bayswater	2	0	2	0	0	0	0	0	0	0
Flushing	1	0	1	0	0	0	0	0	0	0
Forest Hills	4	2	2	0	2	0	34	331	0	10.3
Hammels-Arverne	5	0	5	0	0	0	0	0	0	0
Hunters Point-Sunnyside	13	5	8	4	5	5	37	302	1.7	12.3
Jackson Heights	2	2	0	2	2	2	9	72	2.8	12.5
Jamaica	5	0	5	0	0	0	0	0	0	0
Kew Gardens	2	0	2	0	0	0	0	0	0	0
Lindenwood-Howard Beach	1	0	1	0	0	0	0	0	0	0
North Corona	2	2	0	2	2	5	16	100	5.0	16.0
Ozone Park	5	0	5	0	0	0	0	0	0	0
Queensbridge-Ravenswood4	2	1	1	0	1	0	4	20	0	20.0
Rego Park	1	1	0	0	1	0	8	70	0	11.4
Richmond Hill	3	0	3	0	0	0	0	0	0	0
Ridgewood	5	1	4	1	1	2	11	79	2.5	13.9
South Ozone Park	3	0	3	0	0	0	0	0	0	0
Steinway	2	0	2	0	0	0	0	0	0	0
Woodhaven	3	0	3	0	0	0	0	0	0	0
Woodside	4	3	1	2	3	4	20	215	1.9	9.3
Park-Cemetery-etc.-Queens	1	1	0	0	0	0	0	1	0	0
**Total**	**82**	**26**	**56**	**18**	**25**	**32**	**210**	**1834**		

Alc Ad, Alcohol Advertisement; Vio Ad, Violent Advertisement.

**Table 6:** Frequencies and percentages of advertisements with violent content, including duplicates, observed in the New York City subway system by product/service, and selected aspects of violent imagery

	**N**	**%** ^a^
Product Object		
Movie	441	38.2
TV Show	636	55.1
Play	77	6.7
Public Service	0	0
Animation	304	26.3
Images of 1 or more guns	144	12.5
Weapons other than gun	437	37.9
Images of fighting/attacking	406	35.2
Images of fear	540	46.8
Images of anger/aggression	813	70.5
Images of destruction	227	19.7
Injured people	152	13.2
Words associated with aggression or violence	627	54.3

^a^Percentages based on total number of advertisements with violent content observed, n = 1154.
